# Is the Carli index flawed?: assessing the case for the new retail price index RPIJ

**DOI:** 10.1111/rssa.12061

**Published:** 2014-04-30

**Authors:** Peter Levell

**Affiliations:** Institute for Fiscal StudiesLondon; University College LondonUK

**Keywords:** Inflation, Price indices

## Abstract

The paper discusses the recent decision of the UK's Office for National Statistics to replace the controversial Carli index with the Jevons index in a new version of the retail price index—RPIJ. In doing so we make three contributions to the way that price indices should be selected for measures of consumer price inflation when quantity information is not available (i.e. at the ‘elementary’ level). Firstly, we introduce a new price bouncing test under the test approach for choosing index numbers. Secondly, we provide empirical evidence on the performance of the Carli and Jevons indices in different contexts under the statistical approach. Thirdly, applying something analogous to the principle of insufficient reason, we argue contrary to received wisdom in the literature, that the economic approach *can* be used to choose indices at the elementary level, and moreover that it favours the use of the Jevons index. Overall, we conclude that there is a case against the Carli index and that the Jevons index is to be preferred.

## 1. Introduction

In March 2013, the UK's Office for National Statistics (ONS) started to publish a new inflation index—RPIJ. This index is identical to the long-standing retail price index (RPI), except that it uses a geometric mean of price relatives (known in inflation circles as the *Jevons* index) rather than an arithmetic mean (the *Carli* index) to calculate price changes of goods at the so-called ‘elementary’ level—where expenditures on individual goods are not observed and so only price survey data are used. The Jevons index has long been used in the UK's other measure of consumer price inflation, the consumer price index (CPI). Around the same time as RPIJ was introduced, the United Kingdom Statistics Authority decided that concerns which the ONS had raised about a potential for upward bias in the Carli index meant that the old RPI would no longer be recognized as a national statistic (United Kingdom Statistics Authority, 2013). (To be classified as a ‘national statistic’ published numbers must meet certain standards set out in the ‘Code of practice for official statistics’.) The old RPI is still published but it is now clearly marked with a warning in the ONS's inflation publications.

These decisions have been quite controversial—both among those who do not share the ONS's concerns about the old RPI, and among those who do. The concern for the first group is that the new RPIJ will give a much lower rate of inflation than the RPI. Over the period 1998–2013, the RPI gave an average inflation rate of 2.9% compared with 2.5% that would have been given by RPIJ. The use of the Jevons index in the CPI, and the fact that this tended to mean that it gave a lower measure of inflation than the RPI, has already generated some mistrust in official numbers—particularly as in the last few years the government has replaced the RPI with the CPI for the indexation of state benefits, government pensions and tax thresholds (all measures which are predicted to save the government money). The second group are more concerned by the fact that, having decided that the Carli index is problematic, the ONS opted not to change the RPI, but rather to produce a new index. This was done for the benefit of those who require a consistent series (especially those who struck contracts with the RPI or who own RPI-linked Treasury bonds). For commentators such as Giles (2013), this, however, betrays an important principle that the ONS's ‘statistical methods should be consistent with scientific principles and internationally recognised best practices’ (United Kingdom Statistics Authority, 2009). Giles noted that

‘the ONS has regularly changed the RPI methodology in the past and deleted other series, it could easily have maintained a rump historic derivative of the RPI, calculated on the deficient basis, while changing the main index’.

The ONS probably decided that a change from the Carli to Jevons index was so large that it would in effect create a new index, meaning that the RPI could no longer reasonably be described as a consistent series. Under this line of thinking, previous changes to the RPI were presumably sufficiently small that they arguably just ‘adjusted’ the RPI. However, can we say that an index that has undergone continual adjustment over many years is the same as it was in the beginning, or at some point does it eventually become a new index in any case? The problem of determining whether a series of small changes can turn one index into another is an example of what philosophers call the Sorites paradox (or a ‘little-by-little’ problem). We may think that adding grains of sand one at a time to a pile will eventually make a heap, but, if we also think that adding a single grain of sand cannot make the difference between a heap and a non-heap, then this is in fact impossible. This makes it difficult to decide whether the ONS has already ‘replaced’ the RPI through smaller changes in the past—and so whether it would be justified in replacing it again.

This paper will look at the original reasons for replacing the Carli index. It seems clear that this index has fallen out of favour with national statisticians in the UK, but what are the concerns with this particular index and how should we select index numbers at the elementary level more generally? Did the ONS and United Kingdom Statistics Authority make the right decision? Answering these questions is important not just to decide whether the new RPIJ should have been created in the first place, but also for the more current debate about whether the existing RPI should be considered ‘deficient’ and demoted in the way that it has been. This will require us to delve into the theoretical and practical reasons for preferring one index over another.

The literature on price indices has identified three ways to choose index numbers. In effect, we can ask the following questions.

(a) Does the index respond appropriately when prices change in different situations or does it give answers that we might consider perverse?
(b) Is the index a good *statistical* estimator of the general price change as distinct from relative price changes across goods according to some measure (as we shall discuss below, exactly which measure is a matter of some debate)?
(c) Does the index provide a good measure of how the *cost of living* is changing for consumers (i.e. the costs of obtaining a given level of welfare)?

The first of these is called the *test* approach. This is because we determine which index has the best properties by setting out a list of criteria (‘tests’) and then asking which indices satisfy them. The second is called the *statistical* or stochastic approach. The third is the interpretation of price indices that is used by most economists and as such is referred to as the *economic* approach. The three approaches are essentially separate and can on occasion come to conflicting conclusions. As part of a consultation on the future of the RPI which led to the creation of the new RPIJ, Diewert (2012a) pointed out that the Carli index failed some important axioms of the test approach, that the statistical approach favoured the Jevons index and that the economic approach was inapplicable at the elementary level (when quantity weights were unobserved). These conclusions were essentially endorsed by the ONS and underlay the decision to replace the Carli index in the new RPIJ.

In this paper, we shall explain what each of these approaches is and use them to make our own assessment on the suitability of the Carli index. In doing so we make several contributions not only to the current debate on the new RPIJ but also to the way that elementary indices should be selected more generally. A primary concern of the ONS was the Carli index's sensitivity to so-called price bouncing, which could lead to an upward bias. We formalize these concerns in a new price bouncing test for the test approach. For the statistical approach, we present some evidence on the relative performance of the Carli and Jevons indices. We find no clear evidence for the superiority of one index over the other, and that the relative performances of the Carli and Jevons indices are not invariant to factors such as the month in which the index is calculated; the sample size; the choice of base month against which prices are compared; and the type of goods included in the elementary aggregate. We also argue that the economic approach *can* be applied to the elementary level, and moreover that it favours the Jevons index, by appealing to something analogous to the principle of insufficient reason from information theory. Overall, we conclude that there is a case for replacing the Carli index with the Jevons index.

The remainder of the paper is structured as follows. In Section The retail price index and the consumer price index, we discuss the historical and technical background to the ‘formula effect’ difference between the RPI and CPI. In Sections The test approach, The statistical approach and The economic approach we discuss the test, statistical and economic approaches. Section Conclusion concludes.

The programs that were used to analyse the data can be obtained from

http://wileyonlinelibrary.com/journal/rss-datasets

## 2. The retail price index and the consumer price index

The UK has for a long time been blessed with two headline measures of consumer price inflation—the RPI and the CPI. The RPI is the older of the two, dating back to an ‘interim index’ that was introduced in June 1947 based on an expenditure survey carried out in 1937–1938, and for most of its history was the UK's principal measure of consumer prices. The CPI is the UK's version of the harmonized index of consumer prices, which was developed by the European Union to ensure that member states published comparable measures of inflation.

These two indices differ in several ways which mean that they can give quite different measures of price changes from year to year. For instance, in 2011 CPI growth averaged 4.5% compared with 5.2% for RPI growth. The differences are a result of the data that they draw from, the coverage of the indices and the methods used to calculate average price changes at the so-called elementary level. In recent years, the CPI replaced the RPI for policy purposes, including the uprating of state benefits and pensions and the indexation of tax thresholds. Consequently, the large gap between the two measures, and particularly the factors that mean that the CPI tends to give a lower measure of inflation than the RPI, came increasingly under scrutiny.

Since 2010, the largest factor contributing to the gap between the RPI and CPI has been the differences in the way that price changes are calculated at the lowest level of aggregation in the two indices (which became known as the ‘formula effect’). In October 2012, questions about the formula effect culminated in the opening of a consultation on changes to the methods that are used in the RPI. In January 2013, the consultation concluded that the Carli index ‘did not meet international standards’ (Office for National Statistics, 2012a) and that consequently the new RPIJ would be published in which the Carli index was replaced with the Jevons index.

### 2.1. The formula effect

The formula effect is the difference between the RPI and CPI that results from the different indices that they use at the first stage of aggregation. Aggregation refers to the process by which an overall index such as the RPI or CPI is calculated in successive stages. The calculation of both the RPI and the CPI starts with (essentially the same) sample of prices collected across the country in each month. (There are a few differences. The CPI uses an approach to gathering car prices which is different from that of the RPI.) This sample is then used to produce weighted averages of price changes relative to a base month (in the UK, January; for details, see section The retail price index and the consumer price index of Office for National Statistics (2012b)). In the very first stage, where the ONS does not have expenditure information, an unweighted average of price changes for particular products is taken within different ‘strata’, defined by either region, type of shop (independent or chain retailer) or both. These give what are known as *elementary aggregate* indices. An expenditure-weighted average of these elementary aggregates is then taken to give an overall national average price index for an ‘item’. These different item indices are then aggregated further through expenditure-weighted averages into ‘sections’ or ‘classes’, which are in turn aggregated into ‘groups’. Finally, an overall price index is calculated from the different group indices. Some examples of the ‘goods’ at each stage of aggregation are given in Table 1.

**Table 1:** Examples of goods at different levels of aggregation in the UK

*Level*	*Price*
Elementary aggregate	800 g white unsliced bread sold in the south-east of England
Item	800 g white unsliced bread
Section or class	Bread
Group	Food

There are various indices which can be used to calculate elementary aggregate price changes. These include

(a) the Carli index (Carli, 1804),

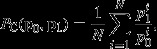
(b) the Dutot index (Dutot, 1738),

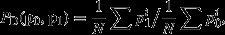
(c) the Jevons index (Jevons, 1865),

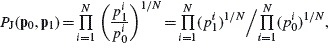
(d) the harmonic index (Coggesshall, 1887) (Diewert (2012a) points out that it was also mentioned earlier in passing in Jevons (1865)),

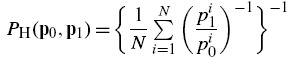
and
(e) the Carruthers–Sellwood–Ward–Dalén (CSWD) index (proposed as an elementary index by Carruthers *et al*. (1980), and also Dalén (1992)),

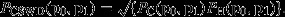

The Carli index is an arithmetic mean of price changes (or price relatives), whereas the Jevons index is a geometric mean. The Dutot index is the ratio of average prices in the base year and the current year. The harmonic index is simply the harmonic mean of price relatives. The CSWD index is a geometric mean of the Carli (arithmetic) and the harmonic indices.

The RPI uses the two arithmetic averages: the Carli and the Dutot indices. The CPI by contrast makes use of the Dutot and the Jevons indices. This puts the CPI closer into line with international practice. Indeed, the RPI's use of the Carli index is quite unusual. None of the other 27 European countries that reported a harmonized index of consumer prices to Eurostat surveyed in Evans (2012) made use of the Carli index in their national price indices. Indeed there seems to be have been a general move away from the Carli index. Evans (2012) listed some countries that have abandoned the Carli index in favour of either the Jevons or the Dutot index over the last few decades including Canada (in 1978), Luxembourg (in 1996), Australia (in 1998), Italy (in 1999) and Switzerland (in 2000). In 1996, the Boskin Commission in the USA recommended that a Carli-like index that was used in the US CPI should be replaced with the Jevons index (Boskin *et al*., 1996)—a change that was put into effect in 1999. Eurostat regulations also do not allow the use of the Carli index in the construction of members’ harmonized index of consumer prices indices except in ‘exceptional cases’ (see section The test approach, page 180, of Eurostat (2001)).

The proportions of elementary aggregates that use each of these formulae in the RPI and CPI are shown in Table 2. For the remaining ‘other’ goods in Table 2, no elementary aggregates are calculated and weights are used at every stage in the calculation of prices. The new RPIJ uses the Jevons in place of the Carli index but continues to use the Dutot index for the same goods as the old RPI. The reason for the even split between the Carli and Dutot index in the old RPI is that both indices can be distorted in particular situations. The Carli index can be too sensitive to situations where individual goods see large price changes (such as when a sale for some items ends). The Dutot index, however, can be dominated by the price movements of a single good, if that good is much more expensive than others included in the calculation (see section 9.3 of Office for National Statistics (2012b)).

**Table 2:** Importance of different formulae used in the RPI and CPI†

*Index*	*Proportion used in the RPI (%)*	*Proportion used in the CPI (%)*
Carli	27	0
Dutot	29	5
Jevons	0	63
Other (weighted) formula	43	33

† Source: Office for National Statistics (2012b).

**Fig 1:**
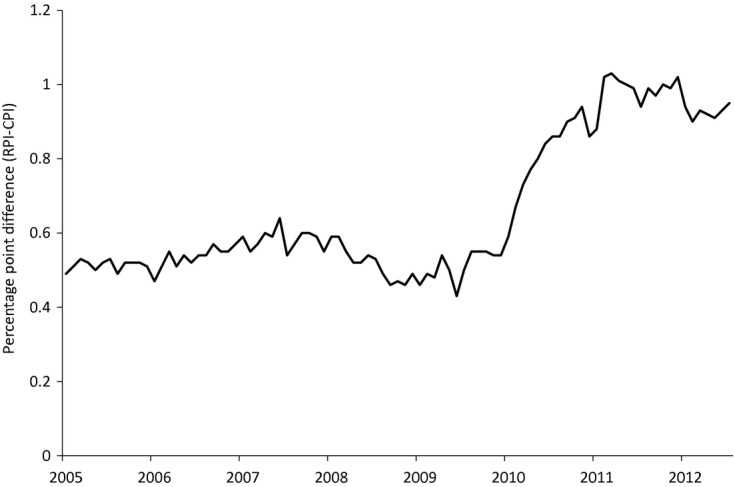
Size of the formula effect, 2005–2012 (source: Office for National Statistics)

What effect do these differences have in practice? Fig. 1 shows the formula effect over time from 2005 to 2012. It shows that the formula effect consistently works to reduce the growth of the CPI substantially relative to the RPI. The effect averaged 0.5 percentage points over the years 2005–2009, which increased to 0.9 since 2010 (for comparison, the average annual increase in the RPI over the same period was 3.4%). The sudden increase in the formula effect can be almost entirely attributed to a change in the sampling of clothing prices that came into effect in that year (Morgan and Gooding, 2010).

This systematic difference is primarily driven by the fact that the Carli and the Jevons indices—being the geometric mean and the arithmetic mean of the price relatives—satisfy the classic inequality

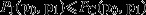
(1)
with equality if and only if 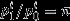 for all *i* (Hardy *et al*. (1934), page 26), i.e. the Jevons index will *always* give either the same or a lower price increase than the Carli index. It is not possible to establish a similar general result for the relationship between the Dutot and the other indices (and thus the formula effect need not necessarily always be positive). Depending on the circumstances the Dutot index could be greater or less than the Carli index and greater or less than the Jevons index. To understand more precisely what drives the formula effect we shall need to delve a littler deeper into the mathematical relationships between the various indices. We do this by presenting the following three facts which will be useful when we turn to evaluating the indices. (Some useful results on the relationships between these and the other indices discussed above by using a theorem from Ladislaus von Bortkiewicz can be found in von der Lippe (2012).) Proofs can be found in the references supplied and are also provided in an on-line annex to this paper.

*Fact 1*: the difference between the Carli index and the Jevons index is bounded from below by the variance of the price relatives (a proof was given in Hardy *et al*. (1934)),

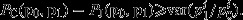

This fact helps to explain the growth in the size of the formula effect in Fig. 1. In 2010, the change to the methods that were used to sample clothing prices led to an increase in the variance of price relatives, which is what led to an increase in the difference between the Carli index used for clothing prices in the RPI and the Jevons index used in the CPI.

*Fact 2*: the difference between the Dutot and the Carli indices equals the covariance of base period prices and price relatives divided by the mean base period price (a proof was given in Carruthers *et al*. (1980)),

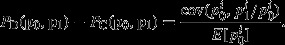
(2)

Finally, by writing the price of the *i*th good in the *t*th period as a multiplicative deviation from its expected value 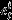 where 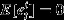


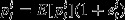
(3)
we can obtain the following useful approximation between the Jevons and Dutot indices.

*Fact 3*: the difference between the Jevons and the Dutot indices depends on the change in the variance of the prices (a result that was first obtained by Carruthers *et al*. (1980); a proof was also given in Diewert (2012a)),

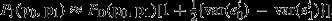


The preceding discussion indicates that the choice of elementary index clearly matters considerably. This makes it all the more important that in any inflation measure the most appropriate indices are chosen—the question to which we now turn. Since there is no definitive set of criteria that we can use to pick one index over another, we try to form judgements applying each of the three approaches used to select elementary indices in the literature (test, statistical and economic). We start with the test approach.

## 3. The test approach

The test approach posits a number of desirable properties for index numbers. These form tests (or ‘axioms’) against which alternative index number formulae can be ranked—with index number formulae which satisfy the most, or the most important, axioms being ranked highest. This approach does not consider any behavioural interdependence between the price and quantity data—unlike the economic approach which we discuss below. The test approach has its roots in the mathematical literature on functional equations, the general problem being that of determining an unknown functional form (i.e. what is the functional form for the price index?) given a set of requirements on the function. The properties are selected to be reasonable given the context.

When we have data on prices and quantities from two periods *t* in {0,1} the problem is to determine the forms of the price index linking the two periods *P*(**p**_0_,**p**_1_,**q**_0_,**q**_1_) and the corresponding quantity index *Q*(**p**_0_,**p**_1_,**q**_0_,**q**_1_) such that the nominal growth rate is (multiplicatively) decomposable in that part reflecting price changes and that part reflecting real changes:

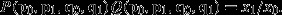


This decomposition property is sometimes called *weak factor reversal* and often is not counted as a ‘test’ but as a defining property of bilateral index numbers. If this holds and neither is 0 then, once we have chosen one index number, the other is chosen implicitly. For example, given a price index we can recover the quantity index implicitly:

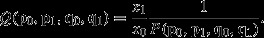


In the case of elementary price aggregates, quantity weights are not observed. Thus the bilateral index number problem is restated slightly as that of finding a price index *P*(**p**_0_,**p**_1_) (and implicitly a quantity index *Q*(**q**_0_,**q**_1_)=(*x*_1_/*x*_0_)/*P*(**p**_0_,**p**_1_)), which satisfies certain tests, such that

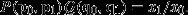


The tests themselves have been developed over the course of well over a century mainly for the case in which prices *and* quantities are observed (for an authoritative discussion of these, see section The retail price index and the consumer price index of Diewert (1992a)). In most cases the tests relating to the price index do not depend on the quantity vectors and so will have obvious analogues for the elementary aggregates case where quantities are not known. (This is not always true. There is, for example, no obvious parallel to the *tabular standard–basket–constant quantities test*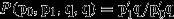 or the *invariance to proportional changes in current quantities testP*(**p**_0_,**p**_1_,**q**_0_,*λ***q**_1_)=*P*(**p**_0_,**p**_1_,**q**_0_,**q**_1_) for *λ*>0 in the context of elementary aggregates. The approach of adapting those tests which are independent of quantities to use for elementary indices was used by Diewert (1995a), who drew on the work of Dalén (1992) and Eichhorn (1978).) We shall divide the tests into four groups: those that simply establish basic properties for an index, those that consider the effects of scalar transformations to prices, a test that bounds the price index and some tests that establish invariance properties. In the rest of this section, we discuss each of these groups in turn. Throughout we assume that 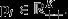 If we want to set **p**_1_=**p**_0_ we call the common vector **p**.

### 3.1. Basic properties

The first set of tests establishes some basic properties which we would expect any price index to have. The first of these is the *positivity test*: we want our price index to be positive for any set of prices, *P*(**p**_0_,**p**_1_)>0 (Diewert (2012b) attributed this to Eichhorn and Voeller (1976)). A non-positive price index would cause all sorts of problems (for instance with chaining). A second basic property is that, if no prices change between two periods, we would expect our price index simply to equal 1, i.e *P*(**p**,**p**)=1. This is the *identity test*, which is sometimes called the constant prices test (Diewert (2012b) pointed out that this test was suggested by Laspeyres (1871), Walsh (1901) and Eichhorn and Voeller (1976)). Finally there are two further tests which establish that certain changes to the prices that we feed into the index should always result in a greater or smaller index. The first of these is the *monotonicity in current prices test* which states that, if we increase one of our current prices, the index as a whole should be greater than it was, i.e. *P*(**p**_0_,**p**_1_)<*P*(**p**_0_,**p**) *if***p**_1_<**p**. Similarly, if we increased any base period price, then the index as a whole should decrease *P*(**p**_0_,**p**_1_)>*P*(**p**,**p**_1_) if **p**_0_<**p** which gives us the *monotonicity in base prices test*. (Here we adopt the notation of Eichhorn and Voeller (1976) for vector inequalities. If *x*=(*x*_1_,*x*_2_, … ,*x*_*n*_) and *y*=(*y*_1_,*y*_2_, … ,*y*_*n*_), then *x*≥*y* if *x*_1_≥*y*_1_,…,*x*_*n*_≥*y*_*n*_ but *x*≠*y*. In other words, all elements of *x* are equal to or greater than those in *y*, with at least one strictly greater.) These last two tests are due to Eichhorn and Voeller (1976) (who actually included these two properties in a single ‘monotonicity’ axiom). Fortunately, all the elementary indices that we mentioned in Section The retail price index and the consumer price index satisfy all four of these tests.

### 3.2. Scalar transformations

The next few tests consider the effects of scalar transformation of the price data. The first two of these demand that the price index should be homogeneous of degree 1 with respect to current prices and homogeneous of degree −1 with respect to base prices, i.e. *P*(**p**_0_,*λ***p**_1_)=*λ* *P*(**p**_0_,**p**_1_) for *λ*>0 and *P*(*λ***p**_0_,**p**_1_)=*λ*^−1^ *P*(**p**_0_,**p**_1_) for *λ*>0. These two requirements are called the *linear homogeneity* and *homogeneity of degree* −1 tests (Eichhorn and Voeller, 1976). These two tests further imply the *dimensionality* test (Eichhorn and Voeller, 1976), which states that the index should not be affected by common changes in scaling to all prices, i.e. *P*(*λ***p**_0_,*λ***p**_1_)=*P*(**p**_0_,**p**_1_). This means among other things that calculating price changes by using pence rather than pounds should make no difference to our indices. The identity and linear homogeneity tests together imply the *proportionality* test (Eichhorn and Voeller, 1976), which states that increasing all prices by a common positive scalar *λ* should give a price index equal to *λ*, or *P*(**p**_0_,*λ***p**_0_)=*λ* for *λ*>0. All our indices satisfy these requirements.

### 3.3. Bound on indices

The next test states that the index itself should fall somewhere in between the price relative of the good with the smallest price increase and the price relative of the good with the largest price increase, or

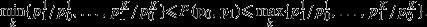

This is the *mean value test* (Eichhorn and Voeller, 1976). Although this last test is a fairly intuitive requirement it can be shown that it is implied by monotonicity in current and base prices, linear homogeneity and identity tests (Eichhorn and Voeller (1976), page 10). Since all our indices satisfy these weaker axioms, they all satisfy this test.

### 3.4. Invariance properties

The final group of tests is concerned with invariance properties of various kinds. This group of tests will help us to discriminate between our three elementary indices and so we shall discuss them in a little more detail.

The first test states that the index should be invariant to the ordering of goods. This implies for instance that, if we took price quotes from the same outlets in a different order (but still keeping the order the same in base and current periods), then this would have no effect on the index.

*Commodity reversal test, or symmetric treatment of outlets*: rearranging the order of the components of both current and base period price vectors in the same way should have no effect on the index, i.e.

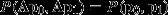

where **A** denotes some permutation matrix which we use to reorder our price vector. Diewert (1992b) attributed this test to Fisher (1922). It can easily be shown that all our indices pass this test. The next test concerns invariance to the units in which goods are defined.

*Commensurability test*: multiplying prices in both periods by a vector *λ* should not affect the index,

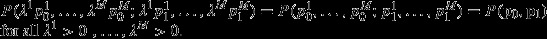


Diewert (1992b) attributed this test to Fisher (1911). This test implies that, ignoring quantity discounts and the like, a change in the units defining an individual item (such as switching from a single item of fruit to a bunch of fruit) should not affect the index. The Dutot index fails this test as it is not in general invariant to changes in the units in which individual goods are sold. If we were to double the base and current period price of one particular item (by, for instance, measuring the price of a pair of gloves rather than a single glove), then the Dutot index would change, whereas our other indices would be unaffected. This comes about because the level of the Dutot index depends on the value of base period prices relative to their mean. (The Dutot index can be rewritten as 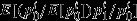 and so can be thought of as an index where price relatives are weighted by base prices.) As Diewert (2012a) pointed out, this means that the Dutot index will not be appropriate for elementary aggregates where there is a large amount of heterogeneity and items are measured in different units, as in these situations ‘the price statistician can change the index simply by changing the units of measurement for some of the items’. However, in most cases goods at the elementary level are typically fairly homogeneous, and so a sleight of hand involving an arbitrary change in the units in which one brand is defined while leaving the definition of other nearly identical products the same may be more easily noticed. If we trust that this is so, we may be satisfied that the index satisfies the weaker dimensionality test that was referred to above (which the Dutot index passes).

The next test states that, if prices go up one period and return to their previous level the next, a chained index should record no price increase.

*Time reversal test*: if the data for the base and current periods are interchanged, then the resulting index is the reciprocal of the original,

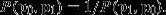


Diewert (1992b) attributed this to Fisher (1922). A sufficient (but not necessary) condition for an index to satisfy this property is that it can be expressed in a form *f*(**p**_*t*_)/*f*(**p**_0_) as it is of course always true that

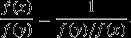


The Jevons and the Dutot indices can be written in this form and so pass the test. The CSWD index cannot but still satisfies time reversal. The Carli and harmonic indices in contrast do not. In fact, if prices go up and then return to their former level the Carli index will record an increase in prices (unless all prices increase in the same proportion), since it can be shown that

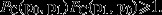

Similarly it can be shown that

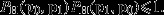

Thus, these indices both fail the time reversal test in a *biased* way. The use of the term bias here is due to Fisher (1922) who used it to refer to a ‘foreseeable tendency [of an index] to err in a particular direction’ (page 86). In this case it refers to the difference between the value of *P*(**p**_0_,**p**_1_) *P*(**p**_1_,**p**_0_) and the ‘correct’ value of 1. It is important to note that it does not refer to bias in the statistical sense of the difference between the expected value of a statistic and its population value. Two indices may both be ‘unbiased’ yet differ considerably in terms of the price changes that they record.

If we then consider price changes over more than two periods, we obtain the following test.

*Circularity test*: the product of a chain of indices over successive periods should equal the total price change over the whole period,

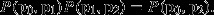


This is a transitivity test. (A related test that is sometimes included is the *multiperiod identity test* that requires that the index satisfies *P*(**p**_0_,**p**_1_) *P*(**p**_1_,**p**_2_) *P*(**p**_2_,**p**_0_)=1 (Diewert (2012b) attributed this to Walsh (1901)). The author is grateful to a referee for demonstrating that this is in fact just an implication of the circularity test.) If this test were not satisfied, then different inflation rates over a given period could be obtained by chaining the index over different subperiods. One consequence of this is that an index could go up or down even if prices had not changed. For instance, consider a case where prices increased from **p**_0_ to **p**_1_ between period 0 and period 1, but in period 2 returned to **p**_0_. In this case, a chained index that did not satisfy circularity could potentially record inflation over the three periods when there had in fact been none.

Eichhorn and Voeller (1976) proved that, unlike in the case of the time reversal test, it is both necessary and sufficient that the index can be written in the form *f*(**p**_*t*_)/*f*(**p**_0_) to pass circularity. The circularity test therefore implies the time reversal test. The Carli index does not satisfy the circularity test since it is 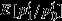 and in general

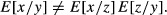


The harmonic and CSWD indices also fail this test.

A final test that we could add to this list concerns so-called price ‘bounces’. This is concerned with how an index would change if different outlets merely exchanged prices from one period to the next. One test of this property, which has been attributed to Dalén (1992), is

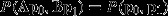

where **A** and **B** are *different* permutation matrices. The Jevons and Dutot indices pass this test, but the Carli, harmonic and CSWD indices fail it. This test has been criticized (Diewert (2012a) for instance called it ‘suspect’) on the grounds that prices should be matched to outlets in a one-to-one manner across periods (i.e. that **p**_0_ and **p**_1_ should not be permuted in different ways), for the simple reason that outlets vary by quality, and so even when the same good is bought in different places it ought to be considered a different product. This is true, but a sensitivity to price bouncing may still be a problem, as it is possible for an index to register a price increase if outlets exchanged prices with one another but then swapped *back*—a property which is somewhat more difficult to justify. Indeed, the problem of the Carli index's sensitivity to price bouncing was highlighted by the ONS in its consultation (Office for National Statistics, 2012c). This concern suggests the following revised test.

*Price bouncing test*: the price index should not change over three periods if prices are just rearranged from the first to the second period and then returned to their original order in the third,

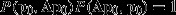

for any possible permutation matrix **A**.

This test will be satisfied by any index that satisfies a stronger property *P*(**p**_0_,**Ap**_0_)=1 (which we may call the strong price bouncing test). This could itself be introduced as a separate test but, by testing how an index would respond to a change which does not match outlet prices across periods, it would be subject to the same criticism as Dalén's price bouncing test. This property is similar to the time reversal test, and indeed it will be satisfied by any index that satisfies time reversal. The two properties are, however, independent as an index may satisfy the price bouncing test but not time reversal. For instance it can be shown that the index

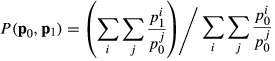

satisfies the price bouncing test, and all our other tests—but not time reversal or circularity. (The author is grateful to a referee for pointing this example out.) The price bouncing test is also independent of its stronger version. Again, we can demonstrate this with an example. Consider the following index, which combines two geometric means of price relatives, weighted by base and current prices:

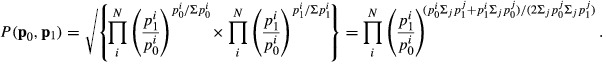


This index satisfies time reversal and so the weak price bouncing test (and indeed all our other tests with the exception of commensurability and circularity) but fails the strong price bouncing test.

The Jevons and Dutot indices satisfy price bouncing as they are in any case invariant to any reordering of prices. Although the CSWD index failed Dalén's old price bouncing test, it passes this new one as it is time reversible. The Carli and harmonic indices, however, fail this test, as we illustrate with a simple numerical example. Table 3 shows how different indices respond to price bouncing in a case with two goods sold in different stores. In period 1 we swap the period 0 prices between store A and store B, and in period 2 we swap them back. In both periods 1 and 2, the Carli index increases by 2.5%, with a cumulative increase over both periods of 5.06%. Similarly, the harmonic index decreases by 2.5% in each period for a cumulative reduction of 4.8%. This is despite prices in period 2 being no different from what they were in period 0! In fact, the Carli index will always show an increase in these sorts of situations and the harmonic index will always show a decrease (for the same reason that in general *P*_C_(**p**_0_,**p**_1_) *P*_C_(**p**_1_,**p**_0_)≥1 and *P*_H_(**p**_0_,**p**_1_) *P*_H_(**p**_1_,**p**_0_)≤1). The Jevons, Dutot and CSWD indices, in contrast, will correctly record no price change, as they do in the example.

**Table 3:** Price bouncing example

*Index*	*Period 0*	*Period 1*	*Period 2*	*Chained index*
Store A price	1	1.25	1	
Store B price	1.25	1	1.25	
		*Period (0,1)*	*Period (1,2)*	
			
Carli	…	(1.25+0.8)/2=1.025	1.025	1.0506
Harmonic	…	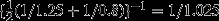	1/1.025	0.952
Dutot	…	1.125/1.125=1	1	1
Jevons	…	√(1.25×0.8)=1	1	1
CSWD	…	√{1.025×(1/1.025)}=1	1	1

Given this list of requirements we can now ask, how do our elementary indices measure up? Table 4 summarizes the results.

**Table 4:** Test performance of the elementary aggregates

*Test*	*Carli*	*Dutot*	*Jevons*	*Harmonic*	*CSWD*
1, positivity	✓	✓	✓	✓	✓
2, identity	✓	✓	✓	✓	✓
3, monotonicity in current prices	✓	✓	✓	✓	✓
4, monotonicity in base period prices	✓	✓	✓	✓	✓
5, linear homogeneity	✓	✓	✓	✓	✓
6, homogeneity of degree −1	✓	✓	✓	✓	✓
7, proportionality	✓	✓	✓	✓	✓
8, dimensionality	✓	✓	✓	✓	✓
9, mean value	✓	✓	✓	✓	✓
10, commodity reversal	✓	✓	✓	✓	✓
11, commensurability	✓	×	✓	✓	✓
12, time reversal	×	✓	✓	×	✓
13, circularity	×	✓	✓	×	×
14, price bouncing	×	✓	✓	×	✓

The Jevons index passes all the tests listed, and the Dutot index fails only the commensurability test. The Carli and harmonic indices fail the time reversal and circularity test and (both) price bouncing tests. The CSWD index fails the circularity test and the original price bouncing test (but not our revised test). Not all the tests are necessarily as important as each other and, in principle, this might present us with an aggregation problem of our own (how to weight the various tests). However, luckily the results are definitive: whatever the weights we place on the individual tests the Jevons index emerges with the strongest axiomatic backing. If we were to consider the importance of the various tests, many would point to the Carli and harmonic indices’ failure of time reversal as being particularly serious. These indices fail this test in a biased manner, meaning that the Carli index for instance will tend to give a higher rate of inflation than other indices that satisfy time reversal. This bias will then be reflected in the price changes that are used in calculations at higher stages of aggregation (as the indices used later satisfy monotonicity), biasing the whole index. Fisher (1922) (page 66) was fairly unequivocal in his condemnation of the Carli index for its failure to satisfy this test, and it was on the basis that the Carli index failed the time reversal test—with an upward bias—that Diewert (2012a) recommended that the Carli index should no longer be used in the RPI. That said, there is not universal agreement on the importance of time reversal. Eichhorn and Voeller (1976) for instance introduced the time reversal and circularity tests by saying that a price index need not ‘necessarily’ satisfy these (the only ‘indisputable’ conditions that a price index should satisfy according to Eichhorn and Voeller (1976) were their monotonicity, linear homogeneity, identity and dimensionality tests—the positivity test was included as part of the definition of a price index). Furthermore, although the Carli index's failure to satisfy time reversibility (and other tests) is indeed a problem, it is important to realize that the RPI and CPI which these elementary aggregates eventually feed into are themselves not time reversible; nor would they be even if the elementary aggregates were time reversible. After the level of the elementary aggregates, the RPI makes use of the Young and Lowe indices to aggregate further. The Young index is not time reversible, and the Lowe index is only time reversible for some comparisons. (For an explanation of these see chapter 1 of International Labor Organization (2004). Diewert (2012a) also recommended that the Young index no longer be used in the RPI.) This means that fixing this particular problem associated with the RPI may not be of that great a benefit, though, as the preceding discussion indicates, it is true that replacing the Carli with a time reversible index would *reduce* any upward time reversal bias of the whole index.

## 4. The statistical approach

The statistical or stochastic approach to index numbers was originally associated with Jevons (1884) and Edgeworth (1925). More recent discussions of the statistical approach can be found in Selvanathan and Prasada Rao (1994), Diewert (1995b) and Clements *et al*. (2006). The statistical approach treats the problem of deciding on the correct price index as an estimation problem. The aim is to separate out a ‘common’ change in prices over two periods (a signal) from relative price changes (which can be considered noise). Different estimator indices can then be evaluated according to the standard statistical considerations of (statistical) bias, and efficiency. The bias of any index is measured against the population object of interest but, unfortunately, there seems to be little agreement in the literature for this approach on what the ‘common’ price change in the population should be.

The ‘unweighted’ stochastic approach aims to estimate the average price change from a population of price relatives 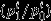. Selvanathan and Prasada Rao (1994) started off by supposing that each price change is made up of a systematic part that is common to all prices and a zero-mean random component *u*^*i*^, so

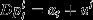
(4)
where 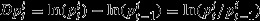 (i.e. the log-increase in prices) and *α*_*t*_ the common trend in all prices which we aim to estimate. The International Labor Organization's consumer price manual (International Labor Organization, 2004) and Diewert (2012a) both used this statistical model to justify the Jevons index. This is because 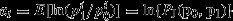 gives the average logarithm of the price changes and taking the anti-logarithm of this gives the Jevons index. However, there are two issues with this conclusion.

Firstly, we can reasonably question whether this is the correct object of interest to consider. A long-standing criticism of the unweighted approach (associated with Keynes (1930) and Walsh (1901)) is that it treats all items equally regardless of their economic importance. Diewert (2012a) for instance referred to this in the context of higher level indices as a ‘fatal flaw’. We might instead think that if for every £1 spent on item A £5 are spent on item B then we should assign B five times the weight of A. This is achieved straightforwardly if we imagine the population to be the price relatives associated with each pound spent rather than associated with each individual item regardless of its price or budget share. This would give us the object of interest 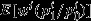 where *w*^*i*^ is the budget share of good *i*, which can also be motivated by the ‘expenditure-based regression model’ in Selvanathan and Prasada Rao (1994).

A second more fundamental issue is that, even if this object of interest is the correct one, the Jevons index does not necessarily give us a good statistical measure in this case. The elementary aggregate price change is 1 plus the percentage increase in prices and the motivation for using 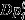 is to make use of the fact that

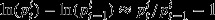

(when growth rates are small). Ultimately our object of interest here is 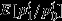. But we know that if this is so then the Jevons index is *biased downwards* (an observation which was first pointed out, in this context, by Greenlees (2001)). This is because Jensen's inequality tells us that *E*[*f*(*x*_*i*_)]≥*f*(*E*[*x*_*i*_]) when *f* is a convex function with equality when all the *x*_*i*_s are the same. When we take the anti-logarithm of *α*_*t*_ we have *f* as the exponential function and 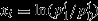 so

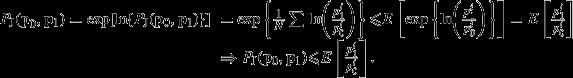


An alternative way to approach this problem is to note that the price relatives themselves can generally (except in some extreme cases) be described by a decomposition into their mean and an additive, mean 0, deviation

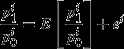
(5)
where *E*[*e*^*i*^]=0 and the variance of *e*^*i*^ is *σ*^2^. We can then estimate 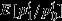 in an unbiased way by taking its sample analogue

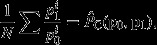

which is the Carli index of price relatives in the sample. Note that this conclusion is not based on any arguments about how price relatives evolve or whether equation (4) is a more realistic model of the process generating price relatives than equation (5). The data-generating process in model (4) implies that current period prices in all outlets would be described by 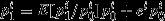, which means that they would equal base period prices inflated by a common factor plus a heteroscedastic deviation. In a process that is described by 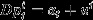, however, log-prices in both periods would be decomposable into a mean and a homoscedastic deviation 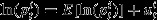 for *t*=0,1 where 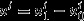. This seems the more realistic model of the way that the data are generated. However, this has no bearing on the question of what the object of interest should be, or on whether the Jevons index is biased as an estimator of 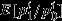.

This need not mean that the use of the Jevons index is ruled out by the unweighted statistical approach, however, as bias is not our only consideration here. The overall performance of an estimator can be summarized by its mean-squared error (MSE), which measures its expected squared deviation from the true population value of the parameter of interest (equal to the sum of its squared bias and its variance), i.e. for some estimator 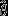 of a population parameter *θ*

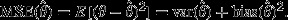
(6)

The sample Jevons index 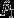 may be a biased estimate of the unweighted population parameter of interest 
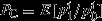
 whereas the Carli index 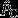 is unbiased, but the Jevons may still perform better than the Carli index in cases where it has a lower variance.

Using equation (6), we can define a measure of the performance of our indices relative to an arbitrary population object of interest *P*. Assuming that we have a statistically random sample, the MSE of the estimator provided by the sample Carli index is simply the variance of the sample mean of price relatives *σ*^2^/*N* plus the Carli index's squared bias

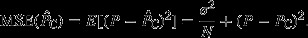
(7)
In the case of the Jevons index, the MSE (using a variance approximation in Dalén (1999), cited in Elliot *et al*. (2012)) is

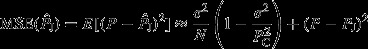
(8)
where 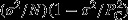 is the approximate variance of the Jevons index. Since it can be shown that 
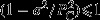
, this means that the approximate variance of the Jevons index will always be smaller—by a constant factor—than the variance of the Carli index.

Combining expressions (7) and (8) tells us that the ratio of MSEs of the Carli and Jevons index for our purposes will be given by

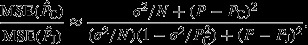
(9)
Expression (9) suggests that it is possible that 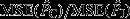 is greater or smaller than 1 depending on the relative biases and variance of the two indices. To say anything further at this point, we need to settle on an appropriate object of interest. The preceding discussion indicates that this would probably be a weighted average of price relatives. We shall adopt period 0 weights in what follows, although one could equally well choose period 1 weights (or some combination of weights in the two periods). This decided, we can now attempt to evaluate the performance of the Carli and Jevons indices in different circumstances.

### 4.1. Empirical exercise

To investigate this, we make use of data from Kantar Worldpanel. This is a panel run by the market research firm Kantar which surveys households throughout Great Britain (Northern Ireland is not included). In this survey, participating households are issued with barcode readers and are asked to scan all barcoded products brought home. In principle this includes groceries purchased from all retailers including on-line outlets, and not just supermarkets. Households also record information on the stores visited. Information on the prices is obtained from till receipts which are mailed to Kantar who then match the prices paid to the purchase record. Where no receipts are available, prices are taken from centralized databases of store- and product-specific prices, or otherwise imputed. The data also record any promotional deal attached to a purchase. Kantar Worldpanel therefore provides us with extremely detailed data on households' expenditure on individual products, including the date that they were scanned and the shop where they were purchased, and whether or not they were on offer. The household purchases that are recorded in the survey give us our own price sample, which we can use for our analysis.

To investigate the statistical performance of the Carli and Jevons indices, we first use the data to construct a ‘population’ of price relatives for different goods. To calculate price relatives for goods seen in different months, we first need to define what we mean by a ‘good’ and then how we decide on its monthly price. We take a good to be a particular product sold in a particular store (products are identified by barcode). Thus two identical loaves of bread sold in two different supermarkets would be considered different goods. The price of goods for a particular month is then taken to be the modal price that consumers paid for 1 unit of this good in that month. The price relative for that month is calculated by deciding on a base month, and then calculating the ratio of prices in the following months relative to that month. We drop any prices that are not observed in every month (leaving us with a balanced panel of prices), which serves to exclude seasonal items that are purchased at only certain times of the year. We also drop the prices of any goods that are on any kind of promotion (such as those subject to quantity deals). The idea behind all these choices is to try to replicate what the ONS or other statistical agency would sample when calculating its elementary aggregates.

To estimate the MSEs of the Carli and Jevons indices, we then employ the following procedure.

*Step 1*: draw a random sample of price relatives of size *n* without replacement from this population.
*Step 2*: calculate sample Carli and Jevons estimators.
*Step 3*: draw another sample, and so on for 30000 iterations.

We repeat this procedure for various sample sizes (for *n*=20,50,80,110,140,170,200), and using different base months. In each case we shall obtain a distribution of sample Carli and Jevons indices which we use to obtain direct estimates of their biases (the average difference between the sample Carli and Jevons indices and the population-weighted mean of price relatives), their MSEs (the average squared difference between the sample Carli and Jevons indices and the population-weighted mean of price relatives) and their variances. We look at the prices of two categories of goods: alcohol and bread. The above selection procedures leave us with 518 price quotes for alcohol in each month and 2319 for bread. Ideally we would like to look at clothing as this is the category of spending for which the formula effect is largest (see Morgan and Gooding (2010)) and therefore the group for which the question of whether the Carli or Jevons index is best statistically is most important. Unfortunately spending on this is not covered by the Kantar Worldpanel which records grocery spending only. Our data cover the months of January–October 2010.

Our work builds on a similar analysis by Elliot *et al*. (2012) who also used alcohol prices in the Kantar Worldpanel covering the years 2003–2011 to look at the statistical performance of various estimators. They found that the MSE of the Carli index tended to be smaller than that of the Jevons index when 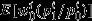 was the object of interest—especially as the sample size increased. (Elliott *et al*. (2012) themselves remained agnostic on what the object of interest should be and considered the performance of different estimators for various target indices.) In very small samples the Jevons index could, however, perform better, suggesting that the statistical approach might favour one or the other in different circumstances. We build on the analysis of Elliot *et al*. (2012) by comparing the performance of the different base months and different times of the year. The interest in the effect of the choice of base month on the two estimators relative to statistical performance stems from its potential to influence the size of the formula effect. Fenwick (1999) pointed out that January sales might increase the variation of prices (especially for goods such as clothing) and, since prices in all subsequent months will then be compared with those in January, these would have a knock-on effect on the variance of price relatives and hence the size of the difference between the Carli and Jevons indices throughout the rest of the year. Different budget shares between goods in different months also mean that the choice of base month may affect the indices' relative biases in our case.

**Fig 2:**
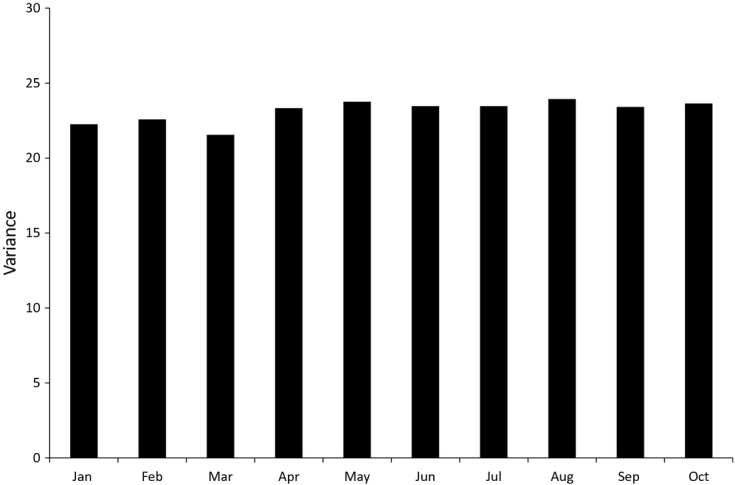
Variation in population modal alcohol prices by month, 2010 (source: author's calculations from Kantar Worldpanel)

The population variance of modal prices in different months for alcohol is shown in Fig. 2. There is little evidence of an effect of January sales in particular on the variance of prices, and the differences from month to month are small. The same is true for the prices of bread which we omit for brevity. This suggests that the channel Fenwick (1999) proposed for differences between the two indices across base months may not be too important in our data. For alcohol the variance is greatest in August (and smallest in March).

**Fig 3:**
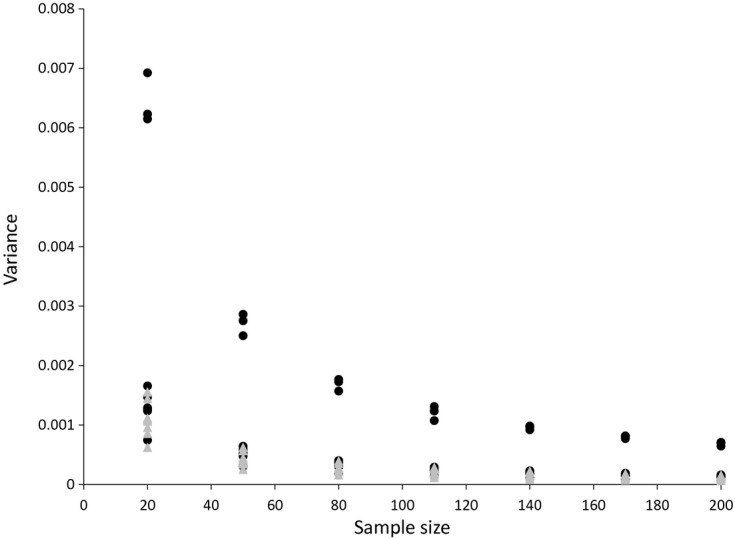
Variance of the Carli and Jevons indices for bread prices (source: author's calculations from Kantar Worldpanel): •, Carli; 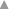, Jevons

The variances of the Carli and Jevons estimators are shown in Figs 3 (for bread) and 4 (for alcohol). These plot the variance for each sample size and each month by using a January base month. The expected relationships hold in that the Jevons index consistently has a lower variance than the Carli index and the variances of both estimators decline approximately with the square root of the sample size.

**Fig 4:**
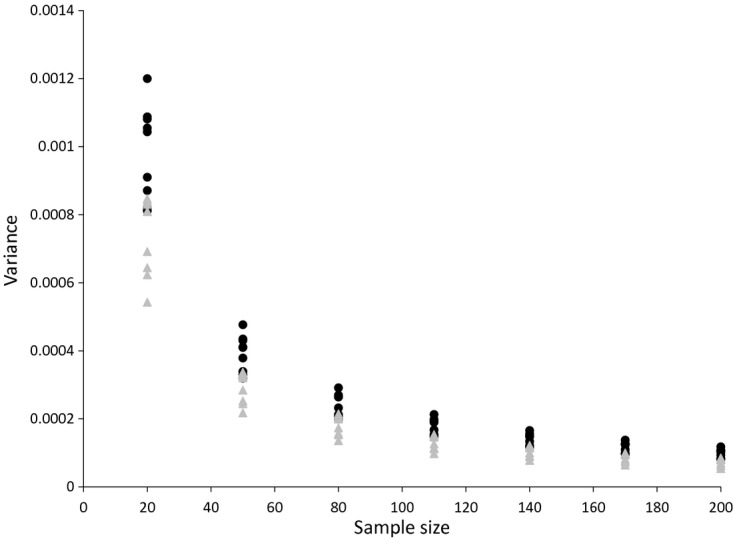
Variance of the Carli and Jevons indices for alcohol prices (source: author's calculations from Kantar Worldpanel): •, Carli; 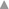, Jevons

**Fig 5:**
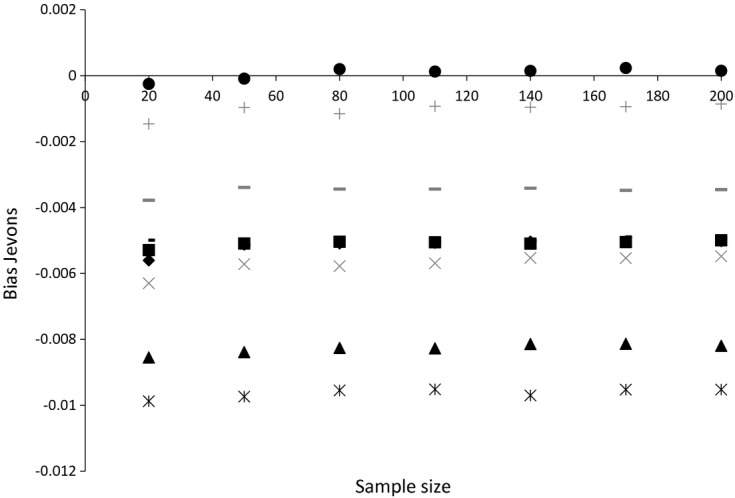
Bias of the Jevons index for alcohol prices (source: author's calculations from Kantar Worldpanel): 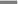, February; 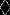, March; 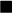, April; ▴, May; ×, June; 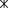, July; •, August; +, September; –, October

The other component of the MSE is the bias. Both the Carli and the Jevons indices are biased estimators for our chosen object of interest, and it is not certain *a priori* which index will be more biased. The Jevons index naturally attaches less importance to the largest price changes, so, if these goods also have the smallest weights, then the Jevons index may be less biased than the Carli index for example. The biases of the Jevons index for different sample sizes for alcohol are shown for each month in Fig. 5 (again using a January base month). It is clear that, unlike the variance, the bias remains roughly constant as the sample size grows. The same is true for the bias of the Carli index for alcohol and the biases of both indices for bread, which for brevity we do not plot. For bread, the Carli index is positively biased for some months and negatively biased for others (March and April), whereas it always has a negative bias for alcohol prices. The Jevons index has a positive bias for bread and a negative bias for alcohol prices (though the bias for alcohol prices is smaller than that of the Carli index).

**Fig 6:**
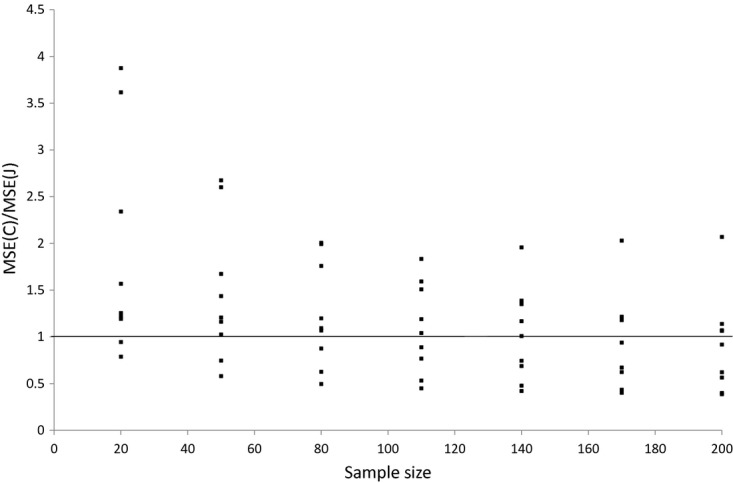
Ratio of Carli and Jevons MSEs for bread by sample size (source: author's calculations from Kantar Worldpanel)

**Fig 7:**
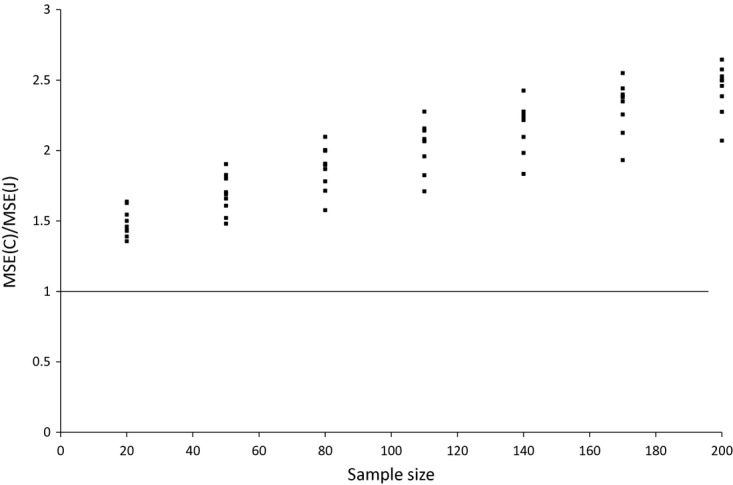
Ratio of Carli and Jevons MSEs for alcohol by sample size (source: author's calculations from Kantar Worldpanel)

We have seen that the Jevons index consistently has a lower variance than the Carli index (which falls with sample size for both estimators) whereas the biases of the two estimators are essentially unaffected by the size of our sample. What does this imply for the two estimators' MSEs? The ratio of the MSE for the two estimators is shown in Figs 6 and 7. For bread the ratio falls as the sample size increases, but the number of months where the Carli index has a greater MSE than the Jevons index is greater only after the sample size exceeds 110. Even then, in some months the Carli index performs much worse than the Jevons index (sometimes with an MSE that is more than twice as high even when the sample size reaches 200). For alcohol the Jevons index seems to perform better in all samples, and the ratio of the MSEs favours the Jevons index as the sample size grows larger. This is because the constant biases have a larger relative effect on the ratio as the variance of the two indices shrinks (and the Jevons index is less biased than the Carli index).

**Fig 8:**
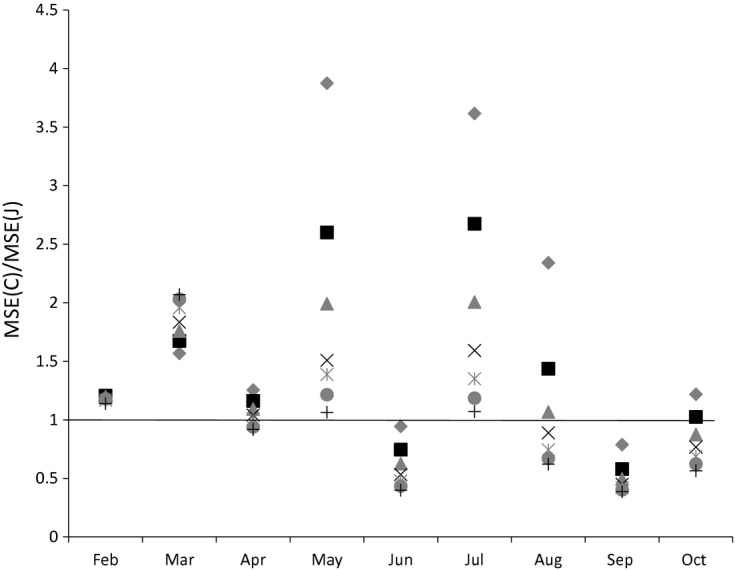
Ratio of Carli and Jevons MSEs for bread by month (source: author's calculations from Kantar Worldpanel): 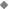, sample size 20; 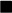, sample size 50; 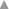, sample size 80; ×, sample size 110; 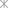, sample size 140; 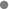, sample size 170; +, sample size 200

**Fig 9:**
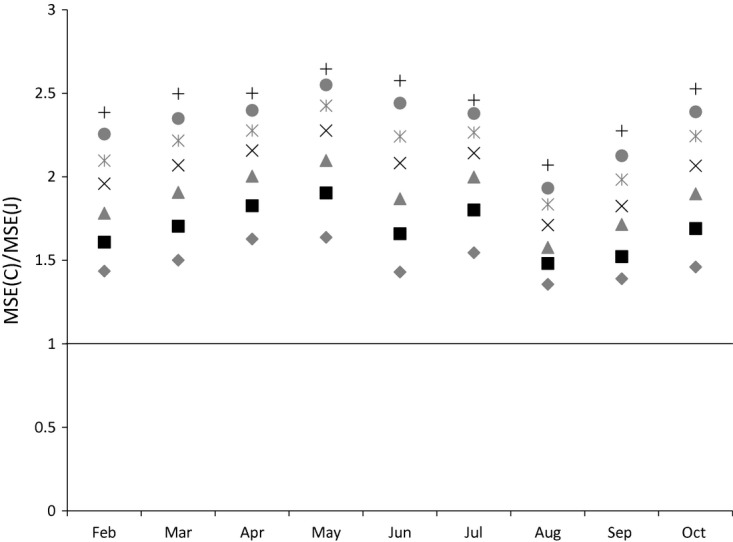
Ratio of Carli and Jevons MSEs for alcohol by month (source: author's calculations from Kantar Worldpanel): 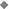, sample size 20; 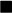, sample size 50; 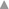, sample size 80; ×, sample size 110; 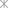, sample size 140; 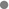, sample size 170; +, sample size 200

We now turn to the question of how the MSEs of the two estimators vary with the time of year. We plot the MSE ratios for various sample sizes in each month for bread and alcohol respectively in Figs 8 and 9 (keeping the base month at January). For alcohol, the Jevons index consistently outperforms the Carli index in terms of MSE in every month. For bread, this can vary. In June, the Carli index has a lower MSE for all sample sizes, whereas in February, May and July the Jevons index always has the lower MSE.

**Fig 10:**
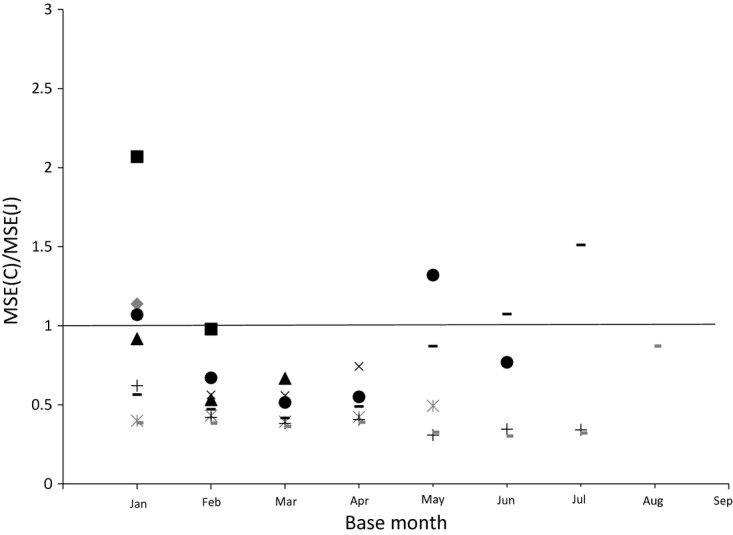
Ratio of Carli and Jevons MSEs for bread by base month (sample size 200) (source: author's calculations from Kantar Worldpanel): 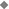, February; 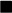, March; ▴, April; ×, May; 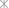, June; •, July; +, August; 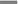, September; 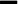, October

**Fig 11:**
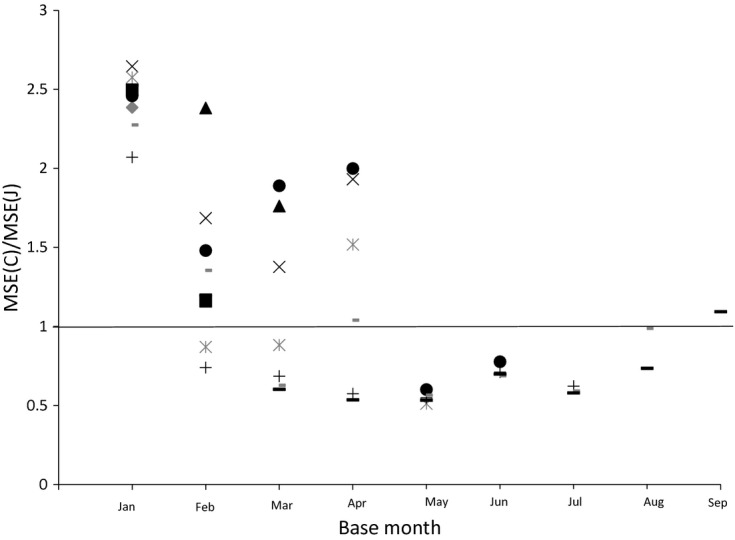
Ratio of Carli and Jevons MSEs for alcohol by base month (sample size 200) (source: author's calculations from Kantar Worldpanel): 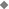, February; 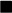, March; ▴, April; ×, May; 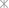, June; •, July; +, August; 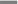, September; 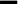, October

A final question concerns the effect of the choice of base month. The Carli–Jevons MSE ratio for different months given different base months is shown for bread in Fig. 10 and for alcohol in Fig. 11. For these plots we keep the sample size constant at 200. We calculate the ratio of MSEs for every month following the base month in the year (so when the base month is September we have price relatives for October only). It is clear that the choice of base month matters. In our data for bread it appears that the case for using the Carli index seems stronger when the base month is February for instance than January. Similarly, for alcohol, the case for using the Jevons index seems much stronger when using a January base month than when we use other base months.

To summarize, the statistical approach does not decisively favour either the Carli or the Jevons index for bread in our data, but the Jevons index consistently outperforms the Carli index for alcohol. This contrasts with the findings of Elliot *et al*. (2012) who found, using data covering a longer time period, that the Carli index tended to perform better as a measure of the population-base-weighted price relatives for alcohol (at least when we use a January base month). We also find that the relative performance of the two estimators varies over the year and according to the base month used. These results have implications for discussions around the size of the formula effect. Since 2010, a change to the methods that are used to collect clothing prices has led to a particularly large difference emerging between the Jevons index used for these goods in the CPI and the Carli index used in the RPI. The changes tended to increase the sample sizes that are used for calculating clothing price relatives. One might have thought that this would favour the Carli over the Jevons index as the variance of the Carli index declines faster with sample size, and indeed this would be true if the object of interest was unweighted. However, with our weighted object of interest, as the sample size increases and the variance decreases, the bias of each index becomes a more important element in determining their relative MSEs. Whether the Carli or Jevons index is more biased for our object of interest depends on which goods see the fastest price increases, and how dispersed the price relatives are. These factors vary over time and across goods, making it difficult to offer any general rules for when the Carli or Jevons index may be more suitable. This suggests caution when trying to generalize results such as those found in Elliot *et al*. (2012).

One potential pitfall with analyses such as these is that they are based on drawing a random sample of price relatives from some population (and implicitly assuming that national statistical agencies would do the same). However, in practice the ONS sample for instance would not appear to be completely random. At present the ONS price sample consists of price quotes for a list of specific items judged in advance to be representative of broader categories: and the selection of these representative items is often a matter of judgement (see Gooding (2012)). The list of representative items is updated annually on the basis of a range of considerations. In 2012 walking or hiking boots replaced outdoor adventure boots as a representative item for footwear. A bag of branded chocolate also replaced candy-coated chocolate as a representative item as its price had been becoming more difficult to collect (for more details see Gooding (2012)). It is not clear how this might affect the relative statistical performance of the sample Carli and Jevons indices. The fact that price relatives are not independently distributed also makes it impossible to calculate reliable standard errors for the elementary aggregates since the actual variance–covariance matrix of *e*^*i*^ in equation (2) will in practice be unknown. This is unfortunate as the prospect of publishing standard errors alongside inflation rates has been mentioned as a key attraction of the statistical approach (see for instance Clements *et al*. (2006)).

## 5. The economic approach

The economic approach to index number construction aims to answer the question: how much more income would a typical consumer require to maintain the same standard of living following a price change? We are typically interested only in maintaining the same ‘economic welfare’ over two periods, by which we mean that we seek only to compensate the consumer for changes in the prices that they face, and not for changes in other environmental factors such as air quality, or changes in the consumer's tastes, which we hold constant for our comparison.

The question is answered conceptually by a cost-of-living index (COLI). The COLI is defined by means of a cost function *c*(**p**_*t*_,*u*_*t*_), which tells us, for any given level of prices **p**_*t*_, the minimum level of expenditure that is needed to achieve a given level of welfare or ‘utility’ *u*_*t*_. The ratio of cost functions in two periods, holding the target utility constant at some level *u*, defines the COLI or Konüs index (dating back to Konüs (1939))

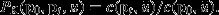


Every household will have its own COLI, and to calculate an economywide inflation measure it is necessary to aggregate these in some way. Various ways of doing this are discussed in Crossley and Pendakur (2010) and aggregation issues are also discussed in Diewert (2001). The COLI is distinct from a cost-of-goods index which conceptually aims to compare the cost of buying a fixed basket of goods in two different periods (rather than to achieve the same utility). The ONS rejects the interpretation of both the CPI and the RPI as attempts to measure changes in the cost of living (see Office for National Statistics (2011)) and instead regards these as cost-of-goods indices.

Under the economic approach, the price index that is chosen should reflect the degree to which consumers mitigate the welfare effect of price changes by shifting their purchases away from goods and services that have become relatively more expensive and towards goods that have become relatively cheaper. This means that it should explicitly take account of the dependence of prices and quantities over time given by the demand function **q**_*t*_(**p**_*t*_). Economists traditionally do this is by representing consumers' decision making (their preferences) with a utility function that ranks different bundles of goods and services, and more importantly is associated with particular demands and particular substitution responses. Each utility function is associated with its own cost function and, if a price index coincides with the ratio of two cost functions for a particular utility function, it can be thought of as representing the COLI for those particular preferences. Two noteworthy price indices that do just this are as follows.

(a) The Laspeyres index

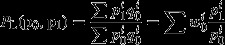

where 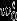 gives the budget shares of good *i* in period 0. This corresponds to the Leontief preferences, where the consumers utility is given by

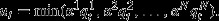

The consumer maximizes this for any given vector of prices by selecting quantities such that

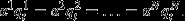

Thus, for Leontief preferences, there are no substitution responses: the ratios of different quantities remain constant as prices change.
(b) The geometric Laspeyres index

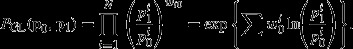

This corresponds to Cobb–Douglas preferences

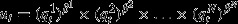

which are in turn associated with the demand functions

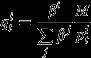

where *M* is the consumer's income. For these preferences, the budget shares will be constant, as 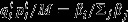, regardless of prices. This implies that a 1% increase in the price of a good results in a 1% reduction in the quantity demanded.

If we think that substitution between products occurs *within* certain groups but not *between* those groups and others (or if preferences within the group are described by Cobb–Douglas or Leontief ‘subutility’ functions), then we could calculate sub-COLIs within groups by using these formulae and then combine them to obtain an overall index in a manner similar to the process of aggregation used to construct the RPI and CPI. For instance, a geometric Laspeyres index could be used within categories of goods where we thought substitution responses were realistically described by Cobb–Douglas preferences, and a Laspeyres index could be used if we thought that it was more appropriate to assume zero substitution.

These indices differ from the unweighted Carli and Jevons and Dutot indices that are actually used in the RPI and CPI, but their resemblance is sometimes used to justify the choice of indices that is used in the calculation of the elementary aggregate price changes. The Jevons index for example is thought to approximate a geometric Laspeyres index within an elementary stratum, and this was one reason behind the Boskin Commission's (Boskin *et al*., 1996) recommendation that the US CPI should make use of the Jevons index for elementary aggregates (when this change was put into effect, the Bureau of Labor Statistics also argued that it would better capture consumers' substitution responses; see for instance Dalton *et al*. (1998)). This kind of logic has, however, been criticized by Diewert (2012a) who writes that

‘… the economic approach cannot be applied at the elementary level unless price and quantity information are both available’.

Since at the level of elementary aggregates such information is not available, it follows that the economic approach should have nothing to say on the subject of which index is preferable.

There are two problems with applying the economic approach when quantities are unknown. The first of these is that, without knowledge of the weights which should be given to each price or price relative, we shall not know whether elementary indices are greater than or smaller than the Laspeyres and geometric Laspeyres indices—in other words the direction and scale of their bias will be unknown. There are in fact particular assumptions about the way that prices are sampled under which elementary indices will equal their COLI counterpart (which are set out in chapter 20 of International Labor Organization (2004)). Most importantly for our purposes, the Carli index will equal the Laspeyres index and the Jevons the geometric Laspeyres index if the price relatives of good *i* are sampled with a probability that is equal to their base period expenditure shares. (The Dutot will equal the Laspeyres index if the probability of sampling good *i* in the base period is equal to the ratio of purchases of *i* in the base period to the total purchases of all goods in *i*'s elementary stratum in the base period.)

These conditions will be true under random sampling in the base period provided that outlets stock goods in proportion to consumers' expenditures on them. However, as we saw in the last section, this assumption is unlikely to hold in practice. If these conditions are not true, and they are essentially impossible to verify, then our elementary indices may end up calculating something rather different from what we intended. Indeed, they may be upwardly or downwardly biased. Thus, unless we had some *reason* to think that the Carli or Dutot index will approximate a true Laspeyres index, or that the Jevons index would approximate a geometric Laspeyres index, then we should be wary about economic justifications for one elementary index over another.

The second problem from a lack of quantity information at this level is that it means that we shall be ignorant of the nature of the interdependence of prices and quantities (consumers' substitution responses), which it is necessary to understand to choose whether our target COLI index should be a Laspeyres or geometric Laspeyres index.

These problems are often used to argue that the economic approach should not be used to choose index numbers at the level of the elementary aggregates, and that different approaches such as the statistical or test approach should be used instead (see for instance Diewert (2012a)). Switching to some other approach is, however, not a particularly satisfying solution. A statistical agency that was employing the economic approach to calculating inflation would still wish to estimate the appropriate COLIs at the elementary level: even if there was insufficient information to construct adequate approximations to these, this does not by itself give us justification to adopt entirely different criteria to select index numbers at this level and this level only. The elementary indices that are chosen for instance by using the test approach could also be greater than or less than what would be suitable given consumers' spending weights and substitution behaviour, and so would be equally problematic. Employing a different approach altogether does not solve the problems that are posed by a lack of information.

Given then that we do seem to have an alternative, how can we select index numbers to approximate consumers' COLIs when we lack all the relevant data? It turns out that, in situations such as these, there is a constructive principle that can be used to guide our choice of index number. This is the principle of maximum entropy (PME), which we now explain.

### 5.1. Principle of maximum entropy

Our problem is that the vectors of budget shares in the base and current periods are unknown at the elementary aggregate level. If we had grounds for selecting one particular vector of base period budget shares for each period from the infinite number of possible combinations for a set of goods, then we could use these to construct indices that approximated either the Laspeyres or the geometric Laspeyres index. If in addition we could select a vector of current period budget shares, then by considering the dependence between shares and prices over time we could also decide which of these two target indices was a more accurate reflection of the COLI for these goods. The question is: why should we choose one particular combination over another? In situations where we have limited knowledge, the PME provides a criterion which we can use to guide our choice of budget shares. This was first proposed by Jaynes in two papers ( Jaynes, 1957a,b) in the context of selecting probability distributions.

To see how this approach works, consider the following example. Suppose that we have a die that has been rolled many times. By ‘many’ we mean a sufficient number for us to ignore any problems of sampling variation. Suppose that the only thing that we are told about these dice rolls is the value of the average roll. What can we say about the probability of rolling a particular number given only this information? This problem would normally be considered insoluble, as Jaynes (1983) noted ‘on orthodox statistical theory, the problem is ill-posed and we have no basis for making any estimate at all’.

Laplace's ‘principle of insufficient reason’ provides us with a first step for assigning probabilities in situations such as these. This states that, in any situation where you want to assign probabilities to different outcomes, you should set them to be equal unless you have reason to do otherwise. The maximum entropy combines the principle of insufficient reason with any information that we do have, and in doing so reflects the idea that we do not want to favour any outcome unless we have adequate justification to do so.

An objective function that will achieve this outcome is the entropy function that was proposed by Shannon (1948):

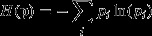

where in the dice example *p*_*i*_ is the probability of rolling number *i*.

This function is maximized when probabilities are uniform and minimized when probabilities are degenerate on a particular outcome. In any given application, we shall want to maximize entropy subject to constraints given by the knowledge that we have (in the dice example, subject to knowledge of the average roll). The constrained optimum to this problem then best represents the current state of knowledge. To choose a distribution with lower entropy than the solution would be to assume information (as measured by Shannon's function) which we do not have. To choose a distribution with higher entropy would violate the constraints provided by the information which we do have from the data. By solving this problem, the maximum entropy approach provides us with estimates of probability distributions in cases where there is insufficient information to use standard statistical methods.

### 5.2. Application of maximum entropy to elementary aggregates

The PME is traditionally applied to situations where we must choose a vector of probabilities. To apply it to our case, we need only to note that the budget shares **w** have all of the necessary properties of probabilities so we can apply the PME to these in the same way. In particular they conform to the Kolomogorov axioms of probability measures (so by definition we can treat them exactly like probabilities).

This suggests the entropy measure

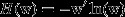

where the budget shares take the place of the probabilities. In the simplest case in which we have no other information (i.e. no constraints aside from the fact that budget shares should sum to 1) the maximum entropy problem is

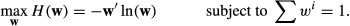
(10)
This is solved by equal budget shares.

*Proposition 1.* The solution to the maximum entropy problem (10) is *w*^*i*^=1/*N* for all *i*.

For a proof of proposition 1, see the on-line appendix.

The intuition behind this solution is as follows. Our problem for selecting budget shares at the level of the elementary aggregates is analogous to the dice problem but in a case where we do not even know the average roll. It seems that we just cannot know what the budget share of each individual good is in the same way as we could not know what the chances of rolling a 1 in the dice example were, which is the reason for rejecting the economic approach. However, just as we can assign some probabilities to dice rolls by using the principle of insufficient reason, we can similarly assign weights by using a budget share equivalent: if we do not have any reason to think that one good should have a greater or smaller budget share than any another, we shall assign them all equal budget shares. (Diewert (2012c) also suggested that an assumption of equal weighting could be used at the elementary level. He used this to justify both the Carli and the harmonic indices as cost-of-goods indices.)

This provides us with a constructive principle which we can use to address our first problem with applying the economic approach to elementary aggregates. The PME justifies the Carli index as an approximation for the Laspeyres index and the Jevons index as an approximation of the geometric Laspeyres index. This is another way of saying that, without additional knowledge, we shall not assign any one good a higher base period budget share than another when calculating our Laspeyres or geometric Laspeyres index.

Our second problem concerned our lack of knowledge of the interdependence of prices and quantities over time. We can proceed in spite of this by noting that the PME can be applied to the vectors of budget shares in *both* periods *t*=0,1. For instance we can solve

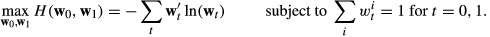
(11)
We can add constraints imposed on the consumer's behaviour by economic theory to this problem. Suppose that we also have available the *total* expenditure on the sum of all of the items in the elementary stratum in each period: denoted {*x*_0_,*x*_1_} where 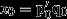 and 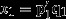. This is the kind of data which may be used to weight elementary aggregates in the next level up. Given this additional data the economic approach to index numbers provides constraints on the budget shares. They must satisfy certain axioms of behaviour provided by the generalized axiom of revealed preference (GARP) (for details see Afriat (1967), Diewert (1973) and Varian (1982)):

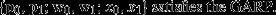

The GARP is a set of inequalities involving the prices, budget shares and total expenditures which provide necessary and sufficient conditions for the standard economic model of consumer choice. (Typically the GARP is applied to prices and quantities but it can easily be rewritten in terms of prices and budget shares since 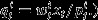 Note that these restrictions are fully non-parametric in the sense that they do not require any knowledge of the consumer's preferences. These constraints can then be added to the maximum entropy problem, which becomes

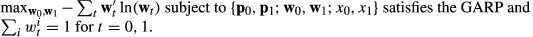
12
The result will be a set of weights which satisfy economic theory and the informational content (as measured by Shannon's index) of the data. We can show that this problem is also solved by equal budget shares in both periods (since this solves the unconstrained problem and it turns out that the restrictions from the GARP are not binding).

*Proposition 2.* The solution to the maximum entropy problem (12) is 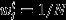 for all *i* and *t*.

For a proof of proposition 2, see Appendix A.

This means that, when you have no data on quantities or budget shares, the PME provides a constructive argument for equal shares across goods and periods of time. These budget shares would be chosen by consumers who had equally weighted Cobb–Douglas preferences. In terms of the choice of elementary index, this would justify the geometric Laspeyres index as a COLI (since this corresponds to the COLI for Cobb–Douglas preferences), and also would justify the Jevons index (since, when budget shares are uniform, an unweighted index will equal the COLI). To choose different vectors of budget shares would assume information which we do not have at this level, and so would not be justified without additional evidence. As we noted before, the PME can also be used to justify the Carli index as an approximation of the Laspeyres index. Thus, the Carli index could also be used in some elementary aggregates if one had some *a priori* grounds for believing that a Leontief utility function (i.e. no substitution) better reflected consumers' preferences. For instance, the Carli index might be more appropriate for elementary aggregates covering pharmaceuticals or goods sold in very different regions.

## 6. Conclusion

Now that we have set out our views on the different approaches to assessing elementary indices, we can ask whether the UK's national statisticians are right to regard the Carli index as flawed and the Jevons index as superior. This after all is the reason underlying both the creation of the new RPIJ and the decision to stop classifying the old (and venerable) RPI as a national statistic. Here we shall sum up our conclusions from the test, statistical and economic approaches and give an overall judgement.

Under the test approach, we noted that the Carli index fails to satisfy various properties which we would expect of a price index, including the important time reversibility test, whereas the Jevons index satisfied all the tests considered. We also find that the Carli index fails a new, revised version of the price bouncing test. It is true, however, that the Carli index's failure to satisfy time reversibility does not provide very strong reasons to replace it in the RPI, an index which is itself not time reversible, and which would not become time reversible if the Carli index were to be replaced (although this would serve to reduce the time reversal bias of the index).

For the statistical approach we noted that we have no *a priori* reason to prefer either the Carli or the Jevons index. Even when our object of interest is an unweighted average of price relatives, the Jevons index, despite its bias, may still have a lower MSE than the Carli index. This is because the Jevons index can have a lower variance than the Carli index. Our view is that it is more appropriate to use the population-weighted price relatives as our target to be estimated. We looked at the relative performance of the Carli and Jevons indices as estimators for this in different contexts for bread and alcohol. This exercise showed that the relative performances of the Carli and Jevons indices are not invariant to considerations such as sample size, the goods included in the elementary aggregate, the base month and the month of the year. This suggests that results found in one context need not necessarily generalize to others.

A common view in the literature is that the economic approach cannot be applied at the level of elementary indices, where quantity information is by definition not available. However, we show that, in the absence of additional information, the PME provides a constructive argument for equal shares across goods and across periods. This approach provides justification for both the Jevons index as an approximation to the geometric Laspeyres index and the Carli index for the Laspeyres index. When applied across periods, the PME suggests use of the geometric Laspeyres index as a target index, as this is consistent with constant budget shares over time. This would favour the use of the Jevons index for elementary aggregates when information on consumers' actual preferences was not available (which will in practice be true for most categories).

Thus, the test and economic approaches both seem to favour the Jevons index over the Carli index, whereas the statistical approach does not provide clear, general guidance. We therefore concur with the general conclusion of the ONS and United Kingdom Statistics Authority that the Jevons index should be preferred to the Carli index.

## APPENDIX

### Appendix A: Proof of proposition 2

This proof consists of two stages. First we show that the solution to the maximum entropy problem (11) is  for all *i* and *t*. Then we show that constant and equal budget shares are consistent with the GARP, and so the additional constraint in this problem is not binding.

The first stage is to solve

which is associated with the Lagrangian

with *λ*_0_ and *λ*_1_ Lagrange multipliers. This has first-order conditions

which as we demonstrate in the proof of proposition 1 implies that  is constant across *i* for both *t*. Combining this with the constraints implies that budget shares will be 1/*N* (constant across both *i* and *t*).

To prove the second stage, we shall show that a violation of the GARP is impossible with equal budget shares. A violation of the GARP implies that there are two periods *t* and *s*, when the consumer chooses quantities *q*_*t*_ and *q*_*s*_ such that  and  or equivalently

and

Our working assumption is that budget shares are constant  for all *i* and *t*. So these conditions imply that

and

Now we know from Jensen's inequality that

since the reciprocal is a convex function. However, since for positive prices and expenditures

then this implies that

which is a contradiction. It follows that equal budget shares must satisfy the GARP.

## Supporting Information

Additional ‘supporting information’ may be found in the on-line version of this article:

‘Online annex to ‘Is the Carli index flawed? Assessing the case for the RPIJ’.
