# Supplementary material for: Is the Carli index flawed?: assessing the case for the new retail price index RPIJ
Source: J R Stat Soc Ser A Stat Soc. 2014 Apr 30;178(2):303–36. doi: 10.1111/rssa.12061 (PMC4309498; doi:10.1111/rssa.12061)
Supplement: Supplementary file 1 — ‘Online annex to ‘Is the Carli index flawed? Assessing the case for the RPIJ’. [file rssa0178-0303-sd1.pdf]

Online Annex to 'Is the Carli index flawed? Assessing the case for the RPIJ'

Peter Levell*

January 20, 2014

Proof. Proof of fact 1.

Using $x_k = \frac{p_1^i}{p_0^i}$, the classical geometric-arithmetic inequality is

$$\prod \left(x^i\right)^{1/N} \leq \sum \frac{1}{N} x^i$$

Using the change of variables

$$x^i = \left(y^i\right)^s$$

and substituting

$$\prod \left[\left(y^i\right)^s\right]^{1/N} \leq \sum \frac{1}{N} \left(y^i\right)^s$$

then taking the sth root gives

$$\prod \left(y^i\right)^{1/N} \leq \left(\sum \frac{1}{N} \left(y^i\right)^s\right)^{1/s}$$

For $s \in (0,1)$ we can use Jensen's inequality again to give

$$\left(\sum \frac{1}{N} \left(y^i\right)^s\right)^{1/s} \leq \sum \frac{1}{N} y^i$$

since z^s is concave with $s \in (0,1)$

$$\sum \frac{1}{N} \left(y^i\right)^s \leq \left(\sum \frac{1}{N} y^i\right)^s$$

Now set $s = 1/2$. Then since $Var\left(X\right) = E\left(X^2\right) - \left[E\left(X\right)\right]^2$

$$\begin{aligned} E\left(X\right) &= \sum \frac{1}{N} \left(y^i\right)^{1/2} \\ \left[E\left(X\right)\right]^2 &= \left(\sum \frac{1}{N} \left(y^i\right)^{1/2}\right)^2 \\ E\left(X^2\right) &= \sum \frac{1}{N} y^i \end{aligned}$$

*Institute for Fiscal Studies and University College London

$$Var\left(X\right)=\sum\frac{1}{N}y^{i}-\left(\sum\frac{1}{N}\left(y^{i}\right)^{1/2}\right)^{2}$$

Using the fact that $\prod\left(y_{i}\right)^{1/N}\leq\left(\sum\frac{1}{N}\left(y^{i}\right)^{s}\right)^{1/s}$(which we have already established) with $s=1/2$ gives

$$\prod\left(y^{i}\right)^{1/N}\leq\left(\sum\frac{1}{N}\left(y^{i}\right)^{1/2}\right)^{2}$$

so

$$Var\left(X\right)\leq\sum\frac{1}{N}y^{i}-\prod\left(y^{i}\right)^{1/N}$$

hence

$$P_{C}\left(\mathbf{p}_{0},\mathbf{p}_{1}\right)-P_{J}\left(\mathbf{p}_{0},\mathbf{p}_{1}\right)\geq Var\left(\frac{p_{1}^{i}}{p_{0}^{i}}\right)$$

□

Proof. Proof of fact 2.

First notice that we can rewrite the Dutot as

$$P_{D}\left(\mathbf{p}_{0},\mathbf{p}_{1}\right)=\frac{E\left[p_{1}^{i}\right]}{E\left[p_{0}^{i}\right]}=\frac{E\left[\left(\frac{p_{1}^{i}}{p_{0}^{i}}\right)p_{0}^{i}\right]}{E\left[p_{0}^{i}\right]}$$

Then notice that the definition of the covariance between p_{0}^{i} and $\left(\frac{p_{i1}}{p_{i0}}\right)$ is

$$Cov\left(p_{0}^{i},\left(\frac{p_{1}^{i}}{p_{0}^{i}}\right)\right)=E\left[p_{0}^{i}\left(\frac{p_{1}^{i}}{p_{0}^{i}}\right)\right]-E\left[p_{0}^{i}\right].E\left[\left(\frac{p_{1}^{i}}{p_{0}^{i}}\right)\right]$$

$$\Rightarrow Cov\left(p_{i0},\left(\frac{p_{1}^{i}}{p_{0}^{i}}\right)\right)/E\left[p_{0}^{i}\right]=E\left[p_{0}^{i}\left(\frac{p_{1}^{i}}{p_{0}^{i}}\right)\right]/E\left[p_{0}^{i}\right]-E\left[\left(\frac{p_{1}^{i}}{p_{0}^{i}}\right)\right]$$

This is just the difference between the Dutot and the Carli, so we have that

$$\Rightarrow P_{D}(\mathbf{p}_{0},\mathbf{p}_{1})-P_{C}(\mathbf{p}_{0},\mathbf{p}_{1})=\frac{Cov\left(p_{0}^{i},\left(\frac{p_{1}^{i}}{p_{0}^{i}}\right)\right)}{E\left[p_{0}^{i}\right]}$$

□

Proof. Proof of proposition 3.

Writing prices as in equation (3), we can think of the Dutot as the empirical counterpart of

$$P_D(\mathbf{p}_0, \mathbf{p}_1) = \frac{E(p_1^i)}{E(p_0^i)}$$

and the Jevons as the counterpart of

$$P_J(\mathbf{p}_0, \mathbf{p}_1) = \prod \left(\frac{E(p_1^i)(1+e_1^i)}{E(p_0^i)(1+e_0^i)} \right)^{1/N} = \frac{E(p_1^i)}{E(p_0^i)} \prod \left(\frac{1+e_1^i}{1+e_0^i} \right)^{1/N}$$

Rearranging gives

$$P_J(\mathbf{p}_0, \mathbf{p}_1) = P_D(\mathbf{p}_0, \mathbf{p}_1) \prod \left(\frac{1+e_1^i}{1+e_0^i} \right)^{1/N}$$

The Jevons is equal to the Dutot multiplied by a function of the deviations in each period. We can approximate the value of $\prod \left(\frac{1+e_1^i}{1+e_0^i} \right)^{1/N}$ by taking a second order Maclaurin expansion.
Let

$$\prod \left(\frac{1+e_1^i}{1+e_0^i} \right)^{1/N} = f(\mathbf{e}_1, \mathbf{e}_0)$$

then our approximation is

$$f(\mathbf{e}_1, \mathbf{e}_0) \approx f(\mathbf{0}, \mathbf{0}) + \begin{bmatrix} \left.\frac{\partial f(\mathbf{e}_1, \mathbf{e}_0)}{\partial \mathbf{e}_1}\right|_{\mathbf{e}_1, \mathbf{e}_0 = 0} & \left.\frac{\partial f(\mathbf{e}_1, \mathbf{e}_0)}{\partial \mathbf{e}_0}\right|_{\mathbf{e}_1, \mathbf{e}_0 = 0} \end{bmatrix} \begin{bmatrix} \mathbf{e}_1 \\ \mathbf{e}_0 \end{bmatrix} + \frac{1}{2} \begin{bmatrix} \mathbf{e}_1' & \mathbf{e}_0' \end{bmatrix} \begin{bmatrix} \frac{\partial^2 f(\mathbf{e}_1, \mathbf{e}_0)}{\partial \mathbf{e}_1 \partial \mathbf{e}_1'} & \frac{\partial^2 f(\mathbf{e}_1, \mathbf{e}_0)}{\partial \mathbf{e}_0 \mathbf{e}_1'} \\ \frac{\partial^2 f(\mathbf{e}_1, \mathbf{e}_0)}{\partial \mathbf{e}_1 \partial \mathbf{e}_0'} & \frac{\partial^2 f(\mathbf{e}_1, \mathbf{e}_0)}{\partial \mathbf{e}_1 \partial \mathbf{e}_1'} \end{bmatrix} \begin{bmatrix} \mathbf{e}_1 \\ \mathbf{e}_0 \end{bmatrix}$$

The derivatives of $f(\mathbf{e}_1, \mathbf{e}_0)$ are the following

$$\left.\frac{\partial f(\mathbf{e}_1, \mathbf{e}_0)}{\partial e_1^i}\right|_{\mathbf{e}_1, \mathbf{e}_0 = 0} = 1/N, \forall i$$

$$\left.\frac{\partial f(\mathbf{e}_1, \mathbf{e}_0)}{\partial e_0^i}\right|_{\mathbf{e}_1, \mathbf{e}_0 = 0} = -1/N, \forall i$$

$$\left.\frac{\partial^2 f(\mathbf{e}_1, \mathbf{e}_0)}{\partial e_1^i \partial e_0^j}\right|_{\mathbf{e}_1, \mathbf{e}_0 = 0} = -(1/N)^2, \forall i, j$$

$$\left.\frac{\partial^2 f(\mathbf{e}_1, \mathbf{e}_0)}{\partial e_1^i \partial e_1^j}\right|_{\mathbf{e}_1, \mathbf{e}_0 = 0} = \left.\frac{\partial^2 f(\mathbf{e}_1, \mathbf{e}_0)}{\partial e_0^i \partial e_0^j}\right|_{\mathbf{e}_1, \mathbf{e}_0 = 0} = (1/N)^2, \forall i \neq j$$

$$\left.\frac{\partial^2 f(\mathbf{e}_1, \mathbf{e}_0)}{\partial (e_1^i)^2}\right|_{\mathbf{e}_1, \mathbf{e}_0 = 0} = \left.\frac{\partial^2 f(\mathbf{e}_1, \mathbf{e}_0)}{\partial (e_0^i)^2}\right|_{\mathbf{e}_1, \mathbf{e}_0 = 0} = \frac{1}{N}(\frac{1}{N} - 1), \forall i$$

So our approximation evaluates to

$$= 1 + \frac{1}{2} \left[\left(\frac{1}{N} \sum e_1^i \right)^2 - \left(\frac{1}{N} \sum (e_1^i)^2 \right) - 2 \left(\frac{1}{N} \sum e_1^i \right) \left(\frac{1}{N} \sum e_0^i \right) - \left(\frac{1}{N} \sum e_0^i \right)^2 + \left(\frac{1}{N} \sum (e_0^i)^2 \right) \right]$$

$$= 1 + \frac{1}{2}\left[\left(\frac{1}{N}\sum(e_0^i)^2\right) - \left(\frac{1}{N}\sum(e_1^i)^2\right)\right]$$

so we have that

$$P_J\left(\mathbf{p}_0, \mathbf{p}_1\right) \approx P_D\left(\mathbf{p}_0, \mathbf{p}_1\right)\left(1 + \frac{1}{2}\left[Var\left(e_0^i\right) - Var\left(e_1^i\right)\right]\right)$$

□

Proof. Proof of proposition 1.

To find the solution to $\max_{\mathbf{w}} -\mathbf{w}' \ln \mathbf{w}$ subject to $\sum w^i = 1$, we first set up the Lagrangian

$$\mathscr{L} = -\mathbf{w}' \ln \mathbf{w} - \lambda(\sum_i w^i - 1)$$

where λ is the Lagrange multiplier. Taking first order conditions gives

$$w^i \frac{1}{w^i} + \ln w^i - \lambda = 0, \forall i$$

$$\implies \ln w^i = \lambda - 1, \forall i$$

$$\implies w^i = \exp(\lambda - 1), \forall i$$

which implies that at the point of maximum entropy, budget shares are constant across i. Combining this with constraint tells us that the entropy maximising budget shares will be $1/N$.

□
